# Detection of structural lesions of the sacroiliac joints in patients with spondyloarthritis: A comparison of T1-weighted 3D spoiled gradient echo MRI and MRI-based synthetic CT versus T1-weighted turbo spin echo MRI

**DOI:** 10.1007/s00256-024-04669-5

**Published:** 2024-04-09

**Authors:** Simon Krabbe, Jakob M. Møller, Anna E. F. Hadsbjerg, Anne Ewald, Stine Hangaard, Susanne J. Pedersen, Mikkel Østergaard

**Affiliations:** 1 Department of Radiology, Herlev and Gentofte Hospital, Borgmester Ib Juuls Vej 1, 2730 Herlev, Denmark; 2 Copenhagen Center for Arthritis Research, Rigshospitalet, Valdemar Hansens Vej 1-27, 2600 Glostrup, Denmark; 3 Department of Clinical Medicine, Faculty of Health and Medical Sciences, University of Copenhagen, Blegdamsvej 3B, 2200 Copenhagen, Denmark

**Keywords:** 3D spoiled gradient echo, Synthetic CT, Spondyloarthritis, Erosion, Sclerosis, Ankylosis

## Abstract

**Objectives:**

To investigate the detection of erosion, sclerosis and ankylosis using 1 mm 3D T1-weighted spoiled gradient echo (T1w-GRE) MRI and 1 mm MRI-based synthetic CT (sCT), compared with conventional 4 mm T1w-TSE.

**Materials and methods:**

Prospective, cross-sectional study. Semi-coronal 4 mm T1w-TSE and axial T1w-GRE with 1.6 mm slice thickness and 0.8 mm spacing between overlapping slices were performed. The T1w-GRE images were processed into sCT images using a commercial deep learning algorithm, BoneMRI. Both were reconstructed into 1 mm semi-coronal images. T1w-TSE, T1w-GRE and sCT images were assessed independently by 3 expert and 4 non-expert readers for erosion, sclerosis and ankylosis. Cohen’s kappa for inter-reader agreement, exact McNemar test for lesion frequencies and Wilcoxon signed-rank test for confidence in lesion detection were used.

**Results:**

Nineteen patients with axial spondyloarthritis were evaluated. T1w-GRE increased inter-reader agreement for detecting erosion (kappa 0.42 vs 0.21 in non-experts), increased detection of erosion (57 vs 43 of 152 joint quadrants) and sclerosis (26 vs 17 of 152 joint quadrants) among experts, and increased reader confidence for scoring erosion and sclerosis. sCT increased inter-reader agreement for detecting sclerosis (kappa 0.69 vs 0.37 in experts) and ankylosis (0.71 vs 0.52 in non-experts), increased detection of sclerosis (34 vs 17 of 152 joint quadrants) and ankylosis (20 vs 13 of 76 joint halves) among experts, and increased reader confidence for scoring erosion, sclerosis and ankylosis.

**Conclusion:**

T1w-GRE and sCT increase sensitivity and reader confidence for the detection of erosion, sclerosis and ankylosis, compared with T1w-TSE.

**Clinical relevance statement:**

These methods improve the detection of sacroiliac joint structural lesions and might be a useful addition to SIJ MRI protocols both in routine clinical care and as structural outcome measures in clinical trials.

## Introduction

Structural damage in the sacroiliac joints is a diagnostic feature of spondyloarthritis. Traditionally, radiography is used to detect structural damage and classify patients as having radiographic versus non-radiographic axial spondyloarthritis by applying the radiographic part of the modified New York criteria. MRI is used to assess ongoing inflammatory disease activity with a fluid-sensitive technique such as short-TI inversion recovery (STIR) or T2-weighted fat-saturated images, and structural damage can be assessed on T1-weighted (T1w) images [1, 2]. However, using low-dose CT of the sacroiliac joints as reference, both radiography and standard T1w spin echo MRI had a limited sensitivity of 42% and 79%, respectively, for detecting erosions [3]. The reliability for detecting structural damage on radiography is limited, likely because it is difficult to assess the complex joints on a single summation image [4]. Furthermore, the reliability for detecting structural lesions using cross-sectional T1w images is often not excellent, even among experts who developed the lesion definitions; recently, the interreader reliability for the absence or presence of each erosive lesion had a mean kappa value of 0.61 in one study, while the intra-class correlation coefficient for a semiquantitative erosion score was 0.69 in another recent study [5, 6]. Images can be perceived differently by two observers, in how the grey-scale voxels of the MRI images are processed into 2D and 3D mental representations of edges and shapes, and insufficient image resolution will decrease lesion detection and interreader reliability, especially for small lesions.

A new method that has been applied to visualize and assess structural lesions of the sacroiliac joints is synthetic CT (sCT) [7–9]. In this method, the patient undergoes an MRI scan with an axial 3D T1-weighted radiofrequency spoiled dual-echo gradient-echo (T1w-GRE) sequence. The images are post-processed with a commercial software that creates CT-like images using a deep learning (artificial intelligence) algorithm, that has been trained on an independent dataset of matching MRI scans and CT scans, to allow it to map individual MRI voxels to estimated Hounsfield units. In a recent study, reconstructed 1 mm semi-coronal sCT led to an improved accuracy for detecting erosion, sclerosis and ankylosis in the sacroiliac joints of axial spondyloarthritis patients, compared with standard semi-coronal 3 mm T1w turbo spin-echo (T1w-TSE) MRI in 2 expert musculoskeletal radiologists [8].

The purpose of this study was to investigate the detection of structural lesions in the sacroiliac joints in patients with spondyloarthritis using 1 mm T1w-GRE and 1 mm sCT, compared with 4 mm T1w-TSE, in a multiple-reader setting with readers having varying levels of experience with assessing sacroiliac joint MRI scans.

## Materials and methods

### Study design and patients

In this cross-sectional study, we included 20 patients who had a clinical diagnosis of axial spondyloarthritis and fulfilled the Assessment of SpondyloArthritis International Society (ASAS) criteria for axial spondyloarthritis [10, 11]. For 1 patient, the MRI images could not be reconstructed into sCT images by the AI algorithm, and therefore 19 patients were included in the analyses. The research was approved by Ethics committee of the Capital Region of Denmark, and all patients gave written informed consent.

### Image acquisition and software processing

Images were obtained at Herlev Hospital, Denmark, using a 3 T Philips Ingenia Elition X MRI scanner. A semi-coronal T1w-TSE sequence was performed with 4 mm slice thickness and 4.4 mm spacing between slices (corresponding to an interslice gap of 0.4 mm). An axial 3D T1-weighted radiofrequency spoiled dual-echo gradient echo (T1w-GRE) sequence was performed with 1.6 mm slice thickness and 0.8 mm spacing between slices (corresponding to 0.8 mm steps with broad overlap between sequential slices, so that any volume is captured by two sequential slices). The T1w-GRE images, using both the in-phase and out-of-phase data, were processed into sub-millimeter 3D sCT image volumes offsite by MRIguidance (BoneMRI V1.4, MRIguidance, Utrecht, Netherlands) using a deep learning-based software that had previously been developed using real CT as reference standard. The axial in-phase T1w-GRE images and the axial sCT images were reconstructed into images in the semi-coronal (coronal oblique) orientation with 1 mm slice thickness using multiplanar reconstruction (Table 1).

**Table 1:** Image parameters for the MRI pulse sequences and the post-processed images

	Semi-coronal T1w-TSE	Axial T1w-GRE	Semi-coronal T1w-GRE	Axial sCT	Semi-coronal sCT
Origin	MRI pulse sequence	MRI pulse sequence	Post-processing: Multiplanar reconstruction from in-phase axial images	Post-processing: Deep learning algorithm applied to axial T1w-GRE	Post-processing: Multiplanar reconstruction from axial images
Orientation	Semi-coronal	Axial	Semi-coronal	Axial	Semi-coronal
Slice thickness	4.0 mm	1.6 mm	1.0 mm	0.8 mm	1.0 mm
Spacing between slices	4.4 mm	0.8 mm	1.0 mm	0.8 mm	1.0 mm
Repetition time	643 ms	7 ms	NA	NA	NA
Echo time	10 ms	2.0 and 3.5 ms	NA	NA	NA
Acquisition time	3 min 3 s	4 min 29 s	NA	NA	NA
Field of view	300 × 180 mm	400 × 240 mm	NA	NA	NA
Matrix	373 × 275	400 × 400	NA	NA	NA
Voxel size	4.0 × 0.8 × 0.7 mm	1.6 × 1.0 × 0.6 mm	NA	NA	NA
Images assessed by the readers	Yes	No	Yes	No	Yes

*T1w-TSE* T1-weigthed turbo spin echo, *T1w-GRE* T1-weighted 3D spoiled gradient echo, *sCT* synthetic CT

### Image assessment

The application of the scoring system was discussed in two initial meetings by the 7 readers. The readers had varying levels of experience in assessing MRI SIJ in research or clinical setting (2 readers > 10 years, 1 reader 5 years, 2 years and 1 year, respectively, and 2 readers were novices (0 years)), as well as varying educational backgrounds (2 rheumatologists, 1 radiologist, 1 research radiographer, 2 radiology trainees and 1 PhD student within spondyloarthritis imaging). The 3 more experienced readers were designated ‘expert readers’ and the 4 less experienced readers were designated ‘non-expert readers’. The non-experts were included to investigate if the results were generalizable to a broader group of readers, which might better reflect potential use in real health care systems. Semi-coronal T1w-TSE images, semi-coronal in-phase T1w-GRE images and semi-coronal sCT images were anonymized and assessed separately and independently by the readers who were blinded to all other images and clinical data. The axial images were not made available to the readers and were not assessed. The three different types of images (T1w-TSE images, T1w-GRE images and sCT images) from the same patient received different id numbers and were randomly placed in three different reading rounds. There was at least 10 days between each reading round. The three different types of images were assessed intermingled and in random order, using an online DICOM viewer that allowed readers to scroll through the images, adjust windowing, zoom and pan, and use a two-point measurement tool. 

Structural lesions were assessed only in the cartilaginous part of the sacroiliac joints. Each joint was divided into 4 quadrants (upper ilium, lower ilium, upper sacrum, and lower sacrum) for scoring of erosion and sclerosis and into 2 halves (upper and lower joint half) for scoring of ankylosis [8]. Each lesion type was judged as either present or absent in each joint quadrant/half, regardless of the number of slices covering the cartilaginous compartment. Erosion on T1w-TSE or T1w-GRE was defined as a bony defect at the joint margin, with full-thickness loss of the dark signal of the subchondral cortex. Similarly, erosion on sCT was defined as a clear interruption of the subchondral cortical bone at the articular surface. Sclerosis was defined as subchondral bands with low-intensity on T1w-TSE or T1w-GRE or high attenuation on sCT extending at least 5 mm from the joint space. Ankylosis on T1w-TSE, T1w-GRE and sCT was defined as bone bridges across the joint with full-thickness loss of the subchondral bone plate on both sides of the joint [8].

Further, the diagnostic confidence of lesions that were judged as present was evaluated using a four-point scale (‘diagnosis’ is used here for each individual lesion type, whereas the patient’s clinical diagnosis was in all cases known a priori by the readers to be axial spondyloarthritis): 1, poor confidence that makes it almost impossible for diagnosis; 2, low confidence that may affect the diagnosis; 3, moderate confidence that does not affect the diagnosis; and 4, high confidence facilitating a clear diagnosis [8]. Again, this was done per quadrant for scoring of erosion and sclerosis and per joint half for scoring of ankylosis,

Reading times for each of the 57 series of images were recorded by each reader from the start of image display until all erosion scores, sclerosis scores and ankylosis scores as well as all diagnostic confidence scores were entered into an electronic scoring sheet.

### Statistics

The inter-reader agreement for judging lesions as present or not (at the joint quadrant level for erosion and sclerosis and at the joint half level for ankylosis) was assessed using the mean of Cohen’s kappa (among all possible reader pairs). Difference in agreement between methods was assessed using bootstrap percentiles for the difference in mean Cohen’s kappa across all reader pairs using 2000 bootstrap samples. Positive findings at the patient level and at the quadrant/joint half level were reported using descriptive statistics and summarized across readers using the median score. Differences in the detection of lesions between imaging methods were compared using the exact McNemar test in 3 pairwise comparisons based on 2 × 2 tables. None of the imaging methods were designated as the reference standard for these analyses. The diagnostic confidence scores for the 3 imaging methods were compared using the Wilcoxon signed-rank test in 3 pairwise comparisons, in which sites with no lesion were included with a confidence score of 0.

### Impression of differences between methods in lesion appearance

After all readers had finished evaluating the cases, one of the readers (SK) performed a detailed pictorial review of selected cases where a lesion had been detected by the readers with moderate or high confidence by all 3 methods, as well as cases where a lesion had been detected by only 1 or 2 methods. The different types of images from the same patient were compared to explore how differences in lesion appearance might explain the observed data patterns.

## Results

### Patient characteristics

All 19 patients had MRI performed within the period of March to November 2021. Mean age was 34 years (range 24–53), body-mass index 25.5 (20–39), time since diagnosis 17 months (1–84), symptom duration 50 months (range 8–110), and 13 were men and 6 women. At the time of the MRI, 16 patients received biological drugs, and the mean Bath Ankylosing Spondylitis Disease Activity Index (a 6-item questionnaire) and the mean pain score were both 4.1 on visual analogue scales from 0 to 10. All patients fulfilled the ASAS criteria for axial spondyloarthritis, 17 had inflammatory back pain, 6 peripheral arthritis, 7 positive family history for spondyloarthritis, 4 psoriasis, 1 inflammatory bowel disease, 3 dactylitis, 4 enthesitis, 1 uveitis, 12 were positive for HLA-B27, 9 had a history of elevated C-reactive protein that was judged related to spondyloarthritis, and 11 fulfilled the radiographic part of the modified New York criteria.

### Reading time

The average reading time for assessing a case and recording the scores into the data entry form was largely similar for the different methods: T1w-TSE 113/145 s (expert/non-expert readers), T1w-GRE 163/164 s, and sCT 150/148 s.

### Inter-reader agreement

The different lesion types were detected with varying levels of inter-reader agreement. In general, inter-reader agreement was higher for experts than for non-experts, as expected (Table 1). Among both experts and non-experts, the highest inter-reader agreement for erosion was found by T1w-GRE, the highest inter-reader agreement for sclerosis was found by sCT, and the highest inter-reader agreement for ankylosis was found by T1w-GRE (Table 2).

**Table 2:** Inter-reader agreement for the presence or absence of lesions

	kappa_T1w-TSE_	kappa_T1w-GRE_	kappa_sCT_	95% CI for difference (kappa_T1w-GRE_ − kappa_T1w-TSE_)	95% CI for difference (kappa_sCT_ − kappa_T1w-TSE_)	95% CI for difference (kappa_sCT_ − kappa_T1w-GRE_)
Erosion, experts	0.43	0.56	0.41	−0.02 to 0.27	−0.19 to 0.15	−0.32 to 0.03
Erosion, non-experts	0.21	0.42	0.39	0.11 to 0.32*	0.08 to 0.29*	−0.16 to 0.10
Erosion, all readers	0.30	0.46	0.41	0.07 to 0.24*	0.01 to 0.20*	−0.17 to 0.06
Sclerosis, experts	0.37	0.53	0.69	−0.03 to 0.35	0.14 to 0.53*	−0.02 to 0.35
Sclerosis, non-experts	0.30	0.39	0.40	−0.03 to 0.23	−0.02 to 0.23	−0.11 to 0.14
Sclerosis, all readers	0.34	0.46	0.50	−0.01 to 0.24	0.03 to 0.28*	−0.08 to 0.15
Ankylosis, experts	0.83	0.88	0.64	−0.12 to 0.22	−0.37 to 0.02	−0.38 to −0.08*
Ankylosis, non-experts	0.52	0.73	0.71	−0.04 to 0.45	−0.03 to 0.41	−0.22 to 0.20
Ankylosis, all readers	0.64	0.79	0.68	−0.04 to 0.34	−0.13 to 0.22	−0.05 to 0.25

Data are based on lesion as assessed at the joint quadrant level (erosion and sclerosis) or at the joint half level (ankylosis). Values are mean kappa value among all possible reader pairs, for 3 experts among 3 reader pairs, for 4 non-experts among 6 reader pairs, for all readers among 21 reader pairs

*CI*, confidence interval; *T1w-TSE*, T1-weigthed turbo spin echo; *T1w-GRE*, T1-weighted 3D spoiled gradient echo; *sCT*, synthetic CT

^*^Significant difference where the 95% confidence intervals do not overlap zero

### Frequency of lesion detection

Erosion was detected more frequently by T1w-GRE compared with T1w-TSE among both experts and non-experts (p = 0.02 and p < 0.001), when analyzed at the joint quadrant level. Also, erosion was detected more frequently by T1w-GRE compared with sCT in both experts and non-experts (p < 0.001 and p = 0.01). No significant differences were found for sCT compared with T1w-TSE. Similarly, at the patient level, erosion was numerically most frequently detected by T1w-TSE or T1w-GRE.

Sclerosis was detected more frequently by T1w-GRE compared with T1w-TSE in experts (p = 0.04), when analyzed at the joint quadrant level, and a similar trend was observed in non-experts (p = 0.09). Also, sclerosis was detected more frequently by sCT than by T1w-TSE in both experts and non-experts (p < 0.001 and p = 0.003). No significant differences were found for sCT compared with T1w-GRE. Similarly, at the patient level, sclerosis was numerically most frequently detected by T1w-GRE or sCT.

Ankylosis was detected with similar frequency by T1w-GRE and T1w-TSE, when analyzed at the joint halves level. Ankylosis was detected more frequently by sCT compared with T1w-TSE in both experts and non-experts (p = 0.02 and p = 0.02). Ankylosis was detected more frequently in sCT compared with T1w-GRE among experts (p = 0.03), but no such trend was observed among non-experts (p = 0.22). Similarly, at the patient level, ankylosis was most frequently detected by sCT (Table 3).

**Table 3:** Frequency of lesion detection

	Number of patients with lesions detected among 19 patients		Number of joint quadrants (erosion and sclerosis) or joint halves (ankylosis) with lesions detected among 152 joint quadrants and 76 joint halves	
	Experts	Non-experts	Experts	Non-experts
Erosion detected by T1w-TSE	17	16	43	53
Erosion detected by T1w-GRE	16	19	57	73
Erosion detected by sCT	14	18	35	59
Sclerosis detected by T1w-TSE	10	17	17	43
Sclerosis detected by T1w-GRE	13	19	26	52
Sclerosis detected by sCT	16	19	34	60
Ankylosis detected by T1w-TSE	4	6	13	15
Ankylosis detected by T1w-GRE	4	4	14	12
Ankylosis detected by sCT	8	9	20	19

Data are the number of patients /quadrants/joint halves with the particular lesion judged as present by at least 2 of 3 expert readers and at least 2 of 4 non-expert readers

*T1w-TSE *T1-weigthed turbo spin echo; *T1w-GRE *T1-weighted 3D spoiled gradient echo, *sCT*, synthetic CT

### Diagnostic confidence scores

**Fig. 1:**
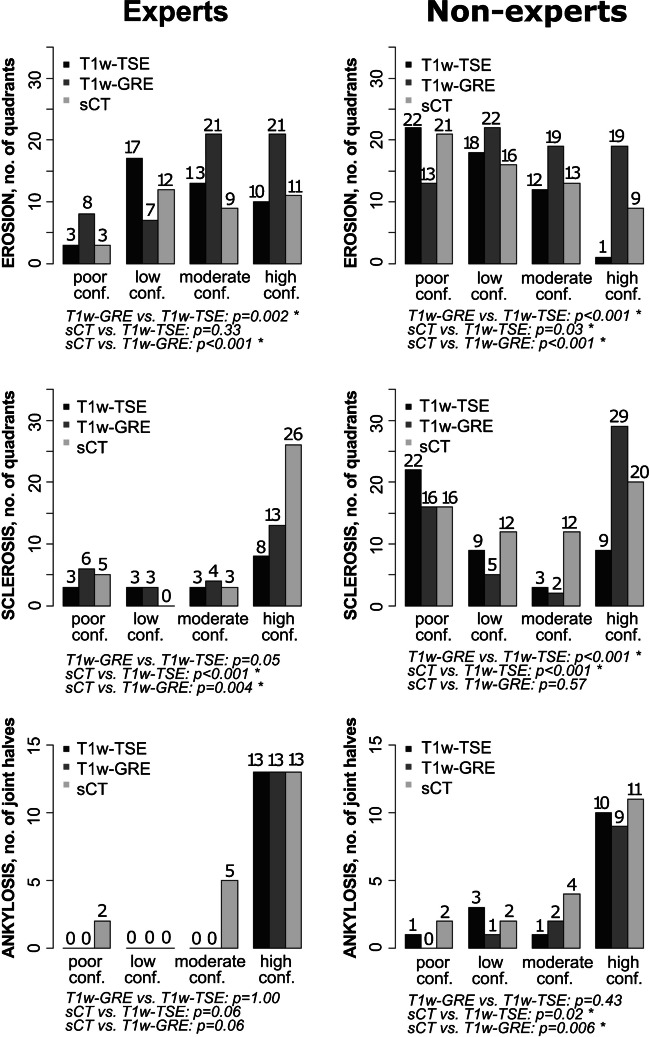
Diagnostic confidence scores. Results are provided as the median confidence score of a lesion being present, as judged by the 3 expert readers and 4 non-expert readers, in 152 joint quadrants for erosion and sclerosis and in 76 joint halves for ankylosis. P-values for pairwise comparisons are Wilcoxon signed-rank test. T1w-TSE, T1-weigthed turbo spin echo; T1w-GRE, T1-weighted 3D spoiled gradient echo, sCT, synthetic CT

The diagnostic confidence scores were in general higher among experts than non-experts, as expected. Different patterns were observed across the lesion types, in that erosion was often detected with only poor, low, or moderate confidence among both experts and non-experts, while sclerosis and ankylosis were mostly detected with high confidence, especially among the experts (Fig. 1).

When comparing the 3 methods, erosion was detected with high confidence most frequently by T1w-GRE in both experts and non-experts. Sclerosis was detected with high confidence most frequently by sCT in experts and by T1w-GRE in non-experts. Ankylosis was detected with high confidence with similar frequency across the methods.

### Impression of differences between methods in lesion appearance

**Fig. 2:**
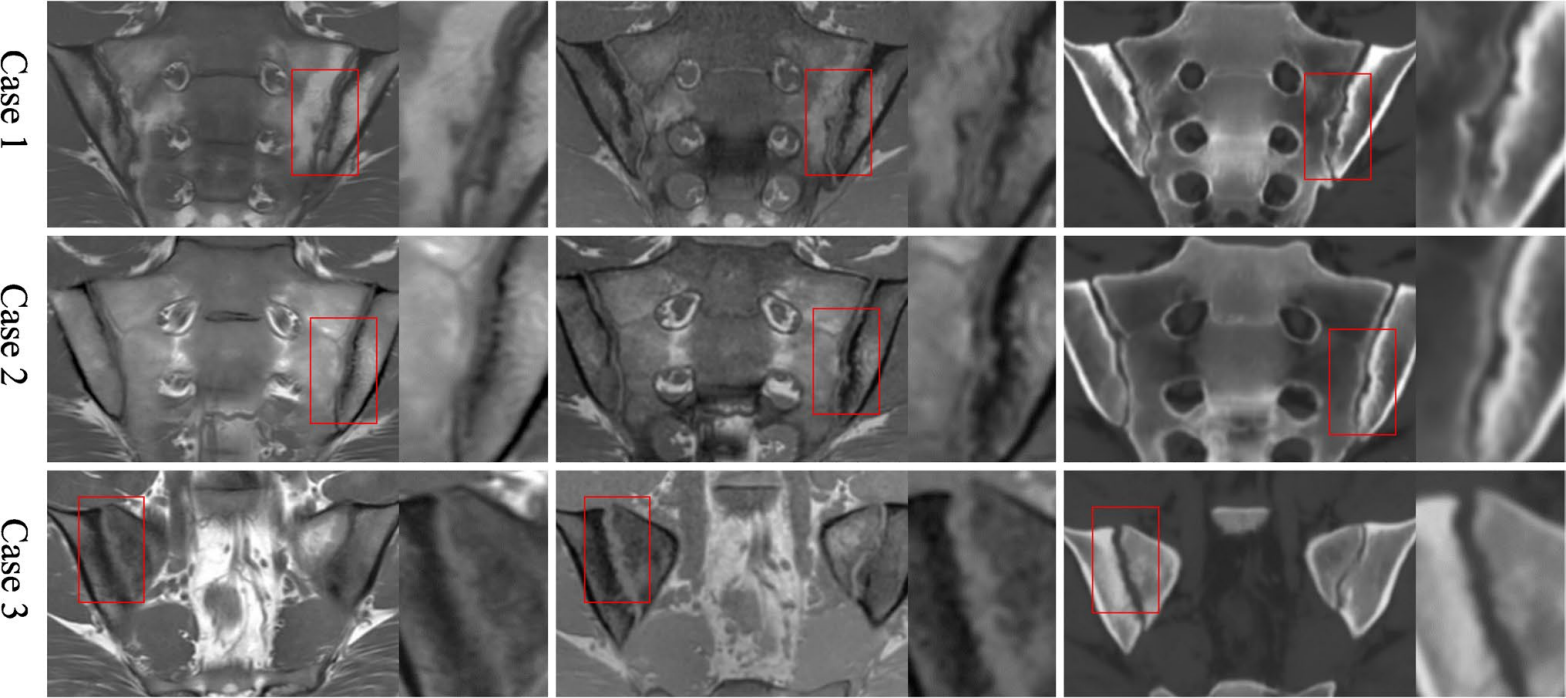
Detection of erosion. T1w-TSE (left images), T1w-GRE (middle) and sCT (right). Case 1: Erosion in the left lower ilium was detected with high confidence by all 3 methods, whereas erosion in the left lower sacrum was only detected by T1w-GRE and sCT. Case 2: Erosion in left lower ilium was clearly demonstrated on T1w-GRE as two adjoined areas of erosion surrounded by thin sclerosis, not detected by T1w-TSE and only detected with low confidence by sCT. Case 3: An extended area of erosion of both the upper and lower sacrum and upper and lower ilium is clearly demonstrated on T1w-GRE with a rugged destructive appearance, less so by T1w-TSE on the iliac side, where it is more blurry, and perhaps also less so by sCT where the appearance is somewhat smoothened on high magnification. Still, the abnormal widening of the right joint space related to extended areas with erosion can easily be appreciated when compared to the normally appearing left joint

It was observed that in cases where erosion was detected on T1w-GRE, but not on T1w-TSE, a more sharply defined, rugged cortical bone could be appreciated on T1w-GRE, with sufficient contrast between cortical bone and erosion cavity, while a less well-defined decrease in signal in the subcortical bone region was apparent on T1w-TSE. Multiple small erosions, confluent in some places, were often surrounded by adjacent sclerosis, as judged from the 1 mm T1w-GRE and sCT, whereas on 4 mm T1w-TSE the increased signal from tissue in erosion cavities and decreased signal from surrounding sclerosis tended to cancel out due to partial volume averaging. Some erosion cavities tended to have a rather smooth appearance in continuity with the surrounding cortical bone on sCT, while they appeared more sharply defined on the T1w-GRE (Fig. 2).

**Fig. 3:**
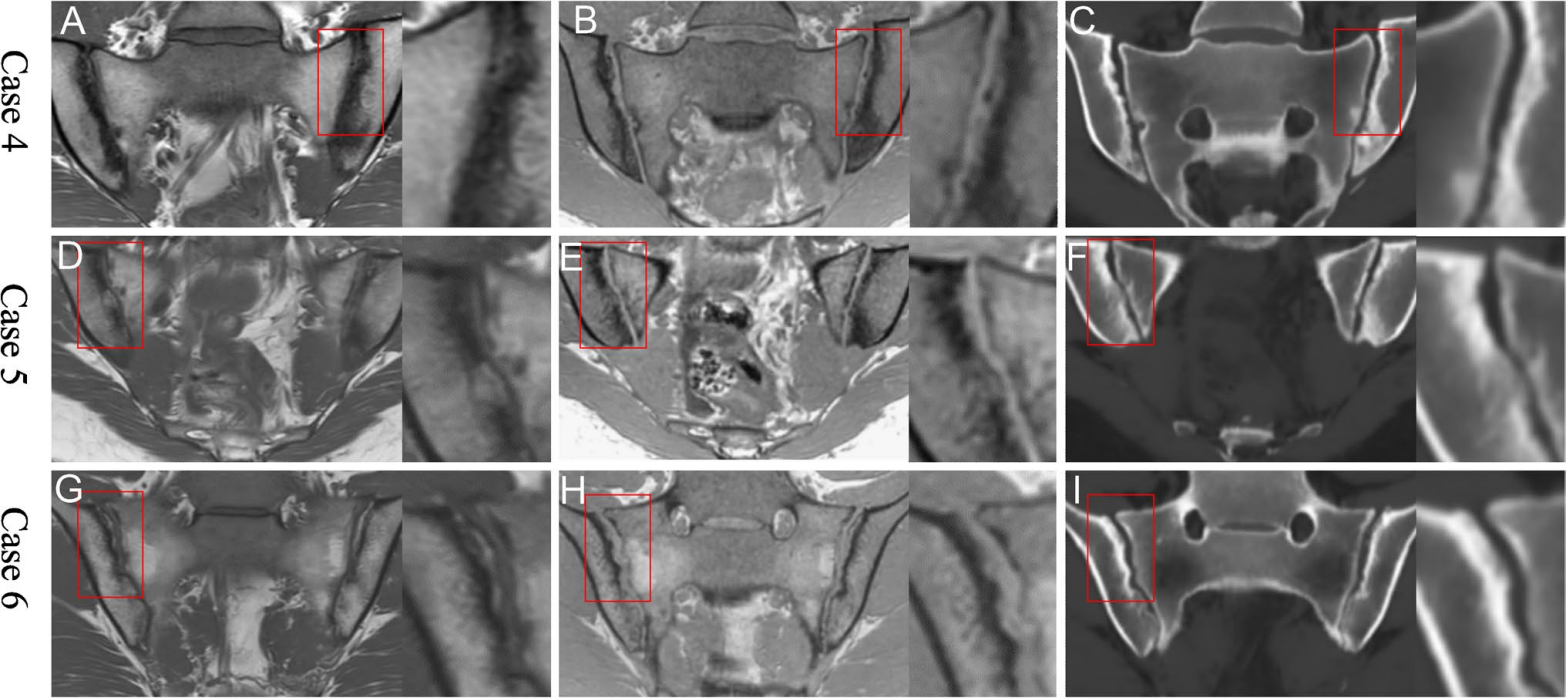
Detection of sclerosis. T1w-TSE (left images), T1w-GRE (middle) and sCT (right). Case 4: Sclerosis in the left upper and lower ilium was detected with high confidence by all 3 methods. Case 5: Sclerosis is clearly seen as a hyperintense layer beneath the cortical bone of the upper right ilium on sCT, and as a corresponding hypointense layer on T1w-GRE, whereas it appears less distinct on T1w-TSE. Case 6: Sclerosis in the upper right ilium may be more readily apparent on sCT than T1w-GRE or T1w-TSE. On T1w-TSE, small areas of fat metaplasia in an erosion cavity (also known as ‘backfill’) is present, and this complimentary information is not seen on the other images

Likewise, in cases where sclerosis was detected on T1w-GRE and sCT, but not on T1w-TSE, a less well-defined decrease in signal in the subcortical bone region was seen on T1w-TSE, compatible with the effect of partial volume averaging. Sclerosis tended to be more sharply defined and therefore easier to see on T1w-GRE and sCT, and it was particularly conspicuous on sCT as bright areas, which may be easier to perceive. On T1w-GRE, areas with sclerosis were hypointense compared with the surrounding normal bone marrow, and attention to fine-tuning the window settings in the DICOM reader is needed to capture these darker areas of sclerosis (Fig. 3).

**Fig. 4:**
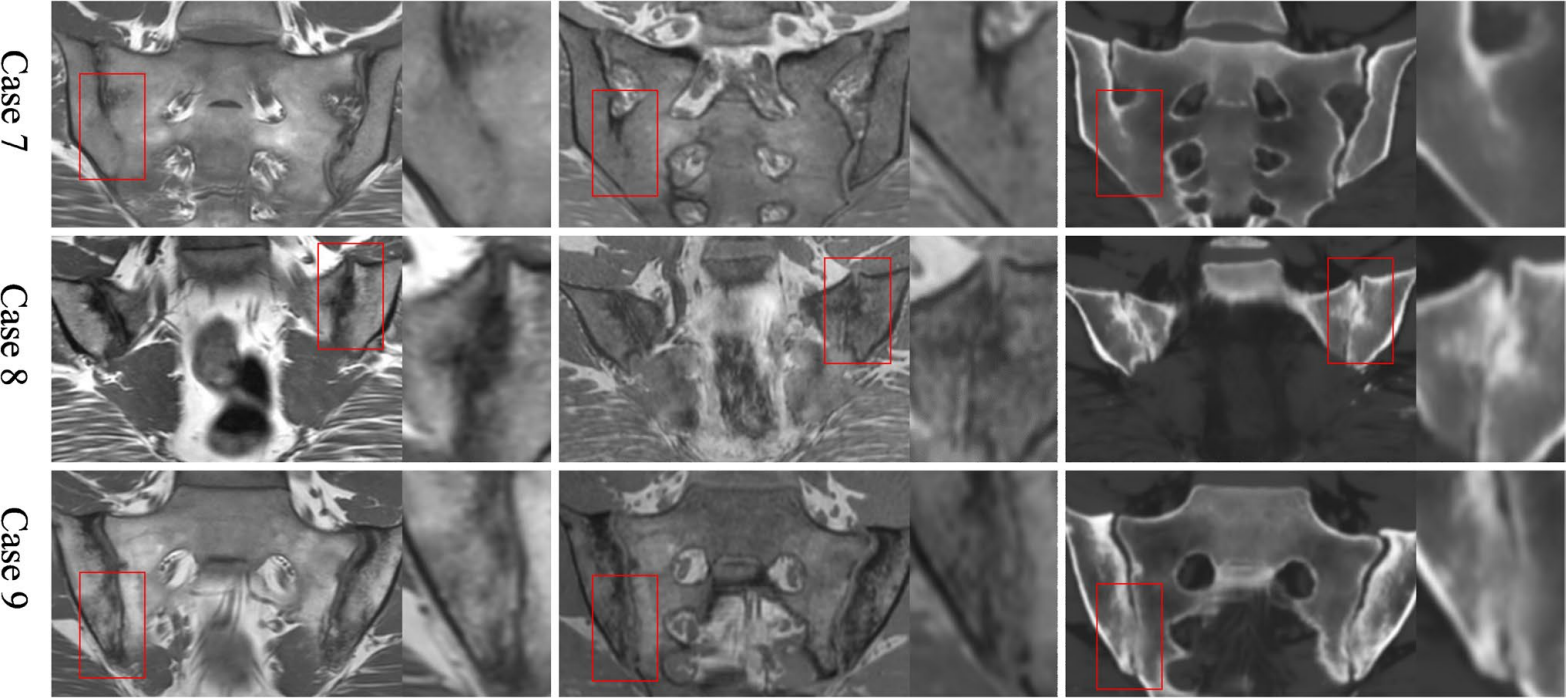
Detection of ankylosis. T1w-TSE (left images), T1w-GRE (middle) and sCT (right). Case 7: Ankylosis of the lower half of right sacroiliac joint was detected with high confidence by all 3 methods. Case 8: Early process of ankylosis on the upper half of the left joint as judged on sCT. On T1w-GRE, a hypointense line that represents cortical bone still separates the two bone marrows, but the joint space appears to be focally absent. On T1w-TSE, a blurry hypointense appearance in this area is difficult to interpret beyond the presence of sclerosis. Case 9: In the lower half of the right sacroiliac joint, the iliac and sacral bones appear to be continuous on sCT, whereas T1w-GRE appears to show more erosion and no definite ankylosis. On T1w-TSE, fat metaplasia in an erosion cavity, also known as ‘backfill’, is present. In this regard, each sequence shows slightly different appearances and the information gained from each may be complementary

Two cases had ankylosis detected by sCT that was not identified by either T1w-GRE or T1w-TSE. Here, in focal areas of one sacroiliac joint, the sacrum and ilium could not be clearly separated on sCT. One of these areas could also be recognized as possible ankylosis on T1w-GRE, when reviewed concurrently with sCT, although less obvious, but not on T1w-TSE. Both may represent an early stage of ankylosis, but definite proof, e.g. with real CT, is not available (Fig. 4).

## Discussion

In this study of patients with axial spondyloarthritis, both 1 mm T1w-GRE and 1 mm MRI-based sCT images of the sacroiliac joints led to improved detection of structural lesions, compared with standard 4 mm T1w-TSE images. Specifically, 1 mm semi-coronal T1w-GRE images of the sacroiliac joints increased the inter-reader agreement for detecting erosion, increased the detection of erosion and sclerosis, and increased reader confidence for scoring erosion and sclerosis. Likewise, 1 mm semi-coronal MRI-based sCT images of the sacroiliac joints, compared with standard 4 mm T1w-TSE images, increased the inter-reader agreement for detecting sclerosis and ankylosis, increased the detection of sclerosis and ankylosis, and increased reader confidence for scoring erosion, sclerosis and ankylosis.

We found that T1w-GRE detected significantly more joint quadrants with erosion than sCT among both experts and non-experts, and confidence scores were higher for T1w-GRE than for sCT. This may be related to a slight blurring of the sCT images, where the junction between the erosion cavity and the surrounding cortical bone in some cases appeared less sharply defined, when these areas were compared side-to-side to the source T1w-GRE images. We speculate that this could reflect a slight loss of spatial information during the post-processing step from T1w-GRE to sCT. Diekhoff et al. suggested that the higher sensitivity of their 0.6 mm thickness VIBE (another spoiled GRE sequence) versus low-dose CT for detecting erosion could be explained by the high contrast on MRI and the image noise on low-dose CT, and that erosions could be missed on low-dose CT while being detected on MRI [12]. Jans et al. found a high accuracy of erosion detection with sCT as compared with low-dose CT as reference standard, but did not assess the T1w-GRE images, which precludes a comparison of T1w-GRE versus sCT in that study [8]. Baraliakos et al. found that their 0.9–1.0 mm thickness VIBE detected more erosions than CT, and also speculated if there was an overcall of erosions on MRI, or whether the T1w-GRE had a better performance than CT [13]. Algin et al. also detected more erosions on a 1 mm thickness 3D-FLASH (another spoiled GRE sequence) compared with 3 mm thickness T1-weighted spin-echo [14]. It is most reasonable to accept that erosions that were judged as present on T1w-GRE were indeed real, and not false positives. The overall distribution of reader confidence scores on T1w-GRE versus sCT versus combined with the sharply defined edges of erosion cavities clearly visualized on T1w-GRE, often in conjunction with adjacent sclerosis, in meticulous side-to-side comparisons between images, seem to support this view.

It should be mentioned that most readers had much less experience with evaluating T1w-GRE and particularly sCT images, compared with T1w-TSE images, and the performance statistics for T1w-GRE and sCT may further improve when readers become more acquainted with these methods.

Part of the reason why erosion and sclerosis were less reliably and less confidently detected on 4 mm images (T1w-TSE), compared with 1 mm images (T1w-GRE and sCT), is probably partial volume averaging. Indeed, besides the already mentioned studies by Diekhoff, Jans, Baraliakos and Algin, a study by Xie et al. found that a 1.2 mm slice thickness VIBE was superior to both a 3 mm slice thickness VIBE and a 3 mm thickness T1w-TSE for erosion detection [15]. Similarly, Chen et al. found that 2 and 3 mm slice thickness T1w images had higher diagnostic accuracy than 4 and 5 mm slice thickness for detecting erosions, using CT as reference standard [16].

sCT detected significantly more joint halves with ankylosis, compared with T1w-TSE in both experts and non-experts. Also, sCT detected significantly more joint halves with ankylosis, compared with T1w-GRE, among experts. However, inter-reader reliability was significantly lower for sCT than T1w-GRE among the experts, and the few additional joint halves in which ankylosis was scored as present by sCT and not by T1w-GRE were only detected with poor or moderate confidence. It appears that in some cases the BoneMRI algorithm was helpful in highlighting sclerosis and ankylosis. This assumes that any artifacts that could mimic sclerosis or ankylosis were not introduced during the post-processing step, since real CT was not available as reference standard.

Overall, this indicates that erosion and sclerosis may be better detected with T1w-GRE, compared with standard T1w-TSE, probably due to the improved slice thickness that is made possible with fast gradient-echo imaging. In addition, the use of the post-processing software that generated sCT images may allow better detection of sclerosis and ankylosis. For practical purposes, sCT images should probably be used in conjunction with the source T1w-GRE images to clarify doubtful findings. Indeed, in the Instructions for Use of the commercial BoneMRI algorithm, it is stated that the reader always needs to inspect both the source images and the sCT images. This mode of assessment was not used in this study. Some structural lesion types defined by the ASAS MRI group were not assessed, specifically bone budding, fat metaplasia in erosion cavities and subchondral fat lesions. T1w-GRE does not obviate the need for acquiring the T1w-TSE to demonstrate fat in the subchondral bone marrow and in erosion cavities as hyperintense signal.

This study had limitations, first, the sample size was 19 patients, which limits the ability to demonstrate significant differences between methods, but despite this, we were able to demonstrate several statistically significant findings that clearly suggest that improved image resolution with T1w-GRE and sCT are promising methods to improve the detection of structural lesions, compared with lower resolution T1w-TSE, in agreement with previous studies [8, 13]. Second, little training was provided to the readers to allow them become acquainted with the different types of images and apply the scoring methodology, and all readers had little experience with the T1w-GRE and particularly sCT images, which may have limited the inter-reader agreement. But strikingly, the inter-reader agreement was significantly higher or tended to be higher for T1w-GRE and sCT (images that were new to all readers) compared with T1w-TSE for most lesion types. Third, the study was not designed to have a reference standard. CT could have been used as such. However, even low-dose CT may be considered inferior to an optimized T1-weighted MRI protocol with sufficient resolution and tissue contrast, and CT was not done since it was not considered necessary for the aims of the current study. In a few joints, ankylosis was detected by sCT with poor or moderate confidence, but not by T1w-GRE. We are unable to prove if this represented true ankylosis, or if it was a false-positive finding on sCT, which would correspond to a higher specificity of T1w-GRE. Fourth, we used the same lesion definitions as Jans et al.,[8] to reproduce the image assessment methodology of that study, well aware that other groups, including the ASAS MRI group, have slightly different wording of the definitions, e.g. the ASAS MRI group also discriminates between erosion and ‘fat metaplasia in an erosion cavity’ (‘backfill’) [6]. This might have led to minor inconsistencies among the expert readers that were mostly used to applying the ASAS definitions, but we consider it unlikely to have had major impact on the comparisons between sequences. Fifth, the study did not attempt to assess the accuracy or confidence in determining the absence of lesions, only their presence. Since no control subjects were included, readers may have had a bias toward detecting lesions with high sensitivity. Our MRI protocol for T1w-TSE had a voxel size of 4.0 mm × 0.8 mm × 0.7 mm, whereas other institutions use a lower slice thickness of 3 mm. In-plane resolution may also differ depending on local protocol optimization. It is possible that a higher resolution T1w-TSE might have increased sensitivity and reader confidence for detecting structural lesions, at the expense of lenghtened scan time. Thus, our results may not be generalizable to centers that use a significantly higher resolution of T1w-TSE. Also, our findings may not be generalizable to newly diagnosed bio-naïve patients, who are likely to have somewhat less damage than the patients included in our study, although we expect the overall trends in lesion detection across imaging methods to be rather similar.

In conclusion, both 1 mm semi-coronal T1w-GRE and 1 mm semi-coronal sCT increase sensitivity and reader confidence for the detection of erosion, sclerosis and ankylosis, compared with standard 4 mm T1w-TSE. These methods might be a useful addition to SIJ MRI protocols both in routine clinical care and as structural outcome measures in clinical trials.

## Data Availability

Data may be shared upon reasonable request.
